# A single gene in *Fusarium oxysporum* limits host range

**DOI:** 10.1111/mpp.13011

**Published:** 2020-11-04

**Authors:** Jiming Li, Like Fokkens, Martijn Rep

**Affiliations:** ^1^ Molecular Plant Pathology University of Amsterdam Amsterdam Netherlands

**Keywords:** cucurbits, effector‐triggered immunity (ETI), *Fusarium oxysoporum* f. sp. *radicis‐cucumerinum*, *Fusarium oxysporum* f. sp. *melonis*, host range

## Abstract

*Fusarium oxysoporum* f. sp. *radicis‐cucumerinum* (Forc) is able to cause disease in cucumber, melon, and watermelon, while *F. oxysporum* f. sp. *melonis* (Fom) can only infect melon plants. Earlier research showed that mobile chromosomes in Forc and Fom determine the difference in host range between Forc and Fom. By closely comparing these pathogenicity chromosomes combined with RNA‐sequencing data, we selected 11 candidate genes that we tested for involvement in the difference in host range between Forc and Fom. One of these candidates is a putative effector gene on the Fom pathogenicity chromosome that has nonidentical homologs on the Forc pathogenicity chromosome. Four independent Forc transformants with this gene from Fom showed strongly reduced or no pathogenicity towards cucumber, while retaining pathogenicity towards melon and watermelon. This suggests that the protein encoded by this gene is recognized by an immune receptor in cucumber plants. This is the first time that a single gene has been demonstrated to determine a difference in host specificity between *formae speciales* of *F. oxysporum*.

## Graphical abstract

A single putative effector gene from melon‐infecting *Fusarium oxysporum* f. sp. *melonis* is able to turn cucumber‐, melon‐ and watermelon‐infecting *F. oxysoporum* f. sp. *radicis‐cucumerinum* nonpathogenic towards cucumber plants.

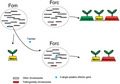

## 1 INTRODUCTION

The *Fusarium oxysporum* (Fo) species complex contains both pathogenic and nonpathogenic strains, and a sexual stage has not been observed so far (Michielse and Rep, 2009; Pietro et al., 2003). As a species complex, Fo can infect more than 120 plant species, including some economically important crops such as banana, cotton, cucumber, and tomato. However, individual isolates can only infect one or a few related plant species (Michielse and Rep, 2009; Pietro et al., 2003). Based on host range, Fo is classified into *formae speciales* (ff. sp.). For example, cucumber, melon, and watermelon‐infecting strains are classified as Fo f. sp. *radicis‐cucumerinum* (Forc), while melon‐infecting strains are classified as Fo f. sp. *melonis* (Fom) (Edel‐Hermann and Lecomte, 2019). Some *formae speciales* are further divided into different races based on host cultivar specificity (Gordon and Martyn, 1997).

To colonize tomato plants, Fo f. sp. *lycopersici* (Fol) secretes proteins as well as enzymes into the xylem, presumably to facilitate infection (Michielse and Rep, 2009; Pietro et al., 2003). So far, 14 small “secreted in xylem” (Six) proteins—also called effectors—from Fol have been identified (Houterman et al., 2007; Michielse and Rep, 2009; Schmidt et al., 2013). Among these effectors, Six1, Six3, and Six5 have been shown to contribute to virulence (Houterman et al., 2009; Ma et al., 2015; Rep et al., 2004; de Sain and Rep, 2015). In contrast, Six2 (Gawehns et al., 2015; Houterman et al., 2007), Six4 (Houterman et al., 2008), Six6, Six9, and Six11 (Vlaardingerbroek et al., 2016b) do not contribute to virulence on a susceptible host under the bioassay conditions tested. Three Six proteins can be recognized by resistance proteins of tomato, triggering an effective immune response. Therefore, these Six proteins are also called avirulence (Avr) proteins. Six4 (Avr1) is recognized by I, and it also suppresses the I‐2‐ and I‐3‐mediated resistance (Houterman et al., 2008; Takken and Rep, 2010). Six3 (Avr2) is recognized by I‐2 (Houterman et al., 2009; Ma et al., 2015), and Six1 (Avr3) is recognized by I‐3 (Rep et al., 2004; Takken and Rep, 2010). This demonstrates that within *formae speciales* effector proteins determine race‐specific resistance in the host. Effectors that trigger host resistance in *formae speciales* in Fo other than Fol have also been identified in Fom (Schmidt et al., 2016) and Fo f. sp. *niveum* (Fon) (Niu et al., 2016).

Because ff. sp. of Fo can be distinguished based on the set of (candidate) effector genes they contain, it seems likely that effector genes contribute to host specificity (Inoue et al., 2017). Until now, individual genes that determine host specificity have not been identified in Fo, but entire chromosomes that determine host range have been identified in Fol (Ma et al., 2010; Vlaardingerbroek et al., 2016a), Forc (van Dam et al., 2017), and Fom (Li et al., 2020). For example, a single chromosome in Forc016, chr^RC^, is responsible for its ability to cause disease in cucumber, melon, and watermelon (van Dam et al., 2017). Fom can only cause disease in melon plants, and chromosome chr^MLN^ determines this host‐specific pathogenicity of Fom (Li et al., 2020). The pathogenicity chromosomes chr^RC^ and chr^MLN^ are highly similar, but the genes on these chromosomes that determine the difference in host range between Forc and Fom have not been identified. One possibility is that (an) extra virulence gene(s) on chr^RC^—absent in chr^MLN^—confer(s) virulence on cucumber and watermelon. Alternatively, a gene on chr^MLN^, but absent on chr^RC^, encodes a protein that can be recognized by an immune receptor of cucumber and watermelon plants, causing nonhost resistance. In this study, we identify a single gene that can explain the difference in host range between Forc and Fom.

## 2 RESULTS

### 2.1 Selection criteria for candidate (a)virulence genes

To identify a gene or genes that are responsible for the difference in host range between Forc and Fom, we set out to find candidate avirulence genes on chr^MLN^ and candidate virulence genes on chr^RC^ by closely comparing the gene sequences of these chromosomes. Based on characteristics of effector genes identified previously in plant‐pathogenic fungi, the following criteria were used for selecting candidate genes: (a) highly expressed in the relevant interaction, because we expect that genes involved in host–pathogen interaction are highly expressed; (b) absent or with less than 100% DNA sequence identity in either f. sp., because we expect that proteins that are not completely identical may have escaped recognition by immune receptors or have altered their function; and (c) presence of a signal peptide for secretion in the predicted translation product, because we assume that effector proteins need to be secreted to function as host‐specific virulence or avirulence factors. We also noted the following characteristics: (a) number of encoded cysteines, because these are important for the stability of secreted proteins in the apoplast; (b) presence of a miniature impala (mimp) element within 2,500 bp upstream of the start codon, because these are commonly found upstream of *SIX* genes in Fo (Schmidt et al., 2013); (c) presence or absence a signal peptide in homologs in other genome sequences; (d) expression level of the homolog in the other *forma specialis*; and (e) predicted function based on similarity with other proteins.

### 2.2 Selection of candidate virulence genes in Forc that could determine host range

To identify genes on chr^RC^ that might be required for cucumber or watermelon infection, the RNA‐sequencing (RNA‐Seq) data generated previously (van Dam et al., 2016) was reanalysed (see Experimental Procedures for details). First, 151 highly expressed genes located on chr^RC^ were selected based on differential expression analysis (http://shiny.imbei.uni‐mainz.de:3838/ideal/). To assess which of these genes are either absent or not identical in Fom, BLASTN was used to detect homologous sequences in the genome assembly of Fom001 (e‐value <10^−20^, perc_identity >90%, query coverage >70%) (van Dam et al., 2017). Twenty genes that are absent in Fom001 were found. We then used SignalP‐5.0 (http://www.cbs.dtu.dk/services/SignalP/) to predict the presence of a signal peptide in the translation products of each of these 20 genes, and found three genes (g287, g288, and g410) to encode potentially secreted proteins. Interestingly, of these three genes, g287 (homolog of *SIX9*) and g288 (unknown function) share a promoter region of about 1 kb and this region includes a mimp. Both have six cysteines in the mature protein sequence. The third gene absent in Fom001, g410, with unknown function, is highly expressed in Forc and also has a mimp upstream of its coding sequence. These three genes were selected as candidate cucumber/watermelon virulence genes.

We also found 35 genes on chr^RC^ with a homolog in Fom001 with sequence identity below 100% (homologous genes). Nine of these were predicted to encode a protein with a signal peptide using SignalP‐5.0. Among these nine genes, g283 and g317 encode the same mature protein with eight cysteines and unknown function, but their homolog in Fom001 is predicted to have no signal peptide. Because these two genes encode the same mature protein, only g317 was selected for further analysis. Among three secreted enzyme genes, g293 and g330—encoding a putative glucosidase 2 subunit β—share 99.7% nucleotide identity, and only g293 was selected for further analysis, while for g291—encoding a putative α‐1,3‐glucosidase—the homolog in Fom001 showed no expression in planta or in vitro and was, therefore, also selected. Lastly, a homolog of *SIX13* (g297) and three genes encoding hypothetical proteins (g250, g310, and g340) were included. The selected 10 candidate Forc‐specific virulence genes are summarized in Table 1. The sequences of the genes and predicted proteins are shown in Table S2.

**TABLE 1:** Virulence and avirulence gene candidates

Candidate virulence genes							
Gene_ID	Mature protein size (amino acids)	Number of cysteines	Mimp or IR	blastn vs. Fom001	SP in Fom homolog	Annotation	Cloned
g287	99	6	Mimp	Absent	‐	Homolog of *SIX9*	Yes
g288	83	6	Mimp	Absent	‐	Unknown	Yes
g410	196	1	Mimp	Absent	‐	Unknown	Yes
g250	132	0	No	96%	Yes	Unknown	Yes
g317	53	8	IR	97%	No	Unknown	NO
g291	938	5	No	98%	Yes	α‐1,3‐glucosidase	Yes
g293	542	16	No	96%	Yes	Glucosidase 2 subunit β	Yes
g297	272	11	Mimp	98%	Yes	Homolog of *SIX13*	Yes
g310	245	8	IR	99%	Yes	Unknown	Yes
g340	150	1	Mimp	95%	Yes	Unknown	Yes

Candidate avirulence genes							
Gene_ID	Mature protein size (amino acids)	Number of cysteines	Mimp or IR	blastn vs. Forc016	SP in Forc homolog	Annotation	Cloned
g14035	132	0	No	98.6%	Yes	Unknown	Yes

Note

GeneID: for candidate virulence genes, the Gene_ID is only based on the annotation of chr^RC^ in Forc016, while for candidate avirulence genes, the gene_ID is from our annotation of this study. Fom, *Fusarium oxysporum* f. sp. *melonis*; Forc, *F. oxysporum* f. sp. *radicis‐cucumerinum*; Mimp, miniature impala; IR, inverted repeats; SP, signal peptide.

### 2.3 Selection of potential avirulence genes in Fom

The difference in host range between Forc and Fom could be determined not only by virulence genes in Forc but also by avirulence genes in Fom. To identify potential avirulence genes for cucumber and/or watermelon in Fom, the Fom001 genome was reannotated using BRAKER_v2.1.0 (see Experimental Procedures) (Hoff et al., 2019). We expected that putative avirulence genes are shared by Fom strains because none of them are able to infect cucumber or watermelon (van Dam et al., 2016). Therefore, we first used BLASTN with 1,284 genes present on the transferable chromosomes of Fom001 (BLASTN: e‐value <10^−20^, perc_identity >99%, and query coverage >70%) against the transferred chromosomes of Fom005 and found 437 shared genes. We expected any avirulence gene for the host range to be either absent or not identical in Forc. Thus, we used BLASTN with the 437 shared genes (e‐value <10^−20^, query coverage >70%) against the Forc016 genome (van Dam et al., 2017). We identified 39 Fom‐specific gene sequences: three genes that are absent and 36 genes with less than 100% identity in Forc016. The presence of a potential signal peptide in the predicted products of these genes was assessed using SignalP‐5.0, and only three were found to encode proteins with a probable signal peptide: a catalase‐peroxidase (g14026), a hypothetical protein (g14035), and a 3‐phytase (g16386). Of these, only g14035 was highly expressed during melon infection (Schmidt et al., 2016). This gene was therefore selected as the only candidate Fom‐specific avirulence gene for further analysis (Table 1). The gene and predicted protein sequences are included as Table S2.

**FIGURE 1:**
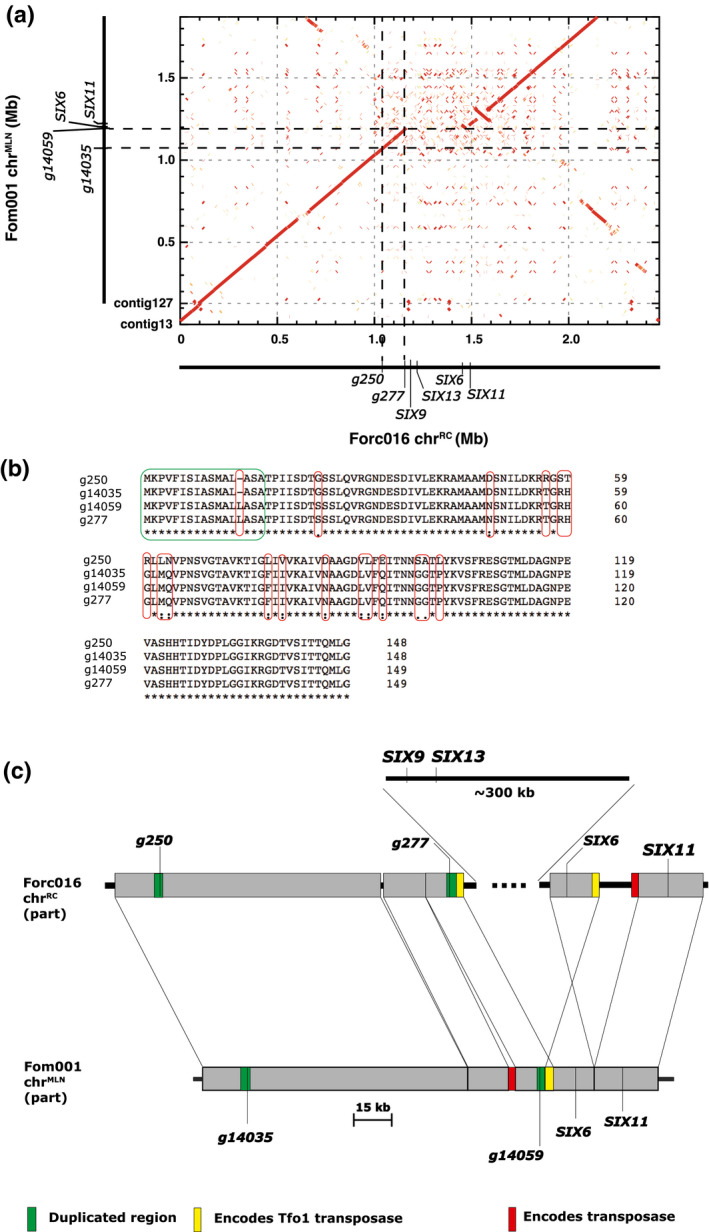
g14035 and its homologs are located in syntenic regions between chr^MLN^ and chr^RC^. (a) g14035, g14059, g250, and g277 are located in syntenic regions of Fom001 chr^MLN^ and Forc016 chr^RC^. The alignment was made with nucmer. (b) Predicted protein sequence alignment of g14035, g14059, g250, and g277. Protein sequences of g14059 and g277 are identical. The predicted mature proteins of g14035 and g14059 only have two amino acids difference, while the predicted protein sequence of g250 is more different from the protein sequence of g14035 (15 amino acids difference). Amino acid differences are marked with red boxes; the predicted signal peptide is marked with a green box. The protein sequences were aligned with Clustal Omega. (c) Comparison of the genomic regions containing g14035 and its homologs on chr^MLN^ and chr^RC^. Around 3.5 kb containing g14035 or its homologs are duplicated on both chr^MLN^ and chr^RC^ (green boxes). A chr^RC^‐specific region of around 300 kb is flanked with a sequence (2,763 bp) encoding a Tfo1 transposase, and this sequence is also present on chr^MLN^ (yellow boxes). Another transposase‐encoding sequence (2,752 bp) is present at different locations on chr^RC^ and chr^MLN^ (red boxes)

Interestingly, a homolog of g14035 in Forc016, g250, is a Forc‐specific candidate virulence gene selected (see above) and is present at a syntenic position in chr^RC^ (Figure 1a). Moreover, there is a homolog of g14035 in Fom001 itself, g14059, also located on chr^MLN^ (Figure 1a). We also found a gene identical in sequence to g14059 in Forc016, g277, located at a syntenic position on chr^RC^ (Figure 1a). The predicted mature proteins of g14035 and g14059/g277 have only two amino acid differences, while the predicted protein sequence of g250 is remarkably more divergent, with 15 amino acids different compared to g14035 (Figure 1b).

To have a better understanding of the genomic regions where g14035 and its homologs are located and the differences between chr^MLN^ and chr^RC^, we manually annotated these regions. Around 3.5 kb, including g250, is duplicated on chr^RC^, and the duplicated sequences share 99% nucleotide identity (green boxes in Figure 1c). Around 3.5 kb containing g14035 is also duplicated (sharing 99% nucleotide identity) on chr^MLN^ of Fom001 (green boxes in Figure 1c). The 3.5 kb containing g277 and g14059 are identical between Forc016 and Fom001 (green boxes in Figure 1c). A large region (around 300 kb) containing *SIX9* and *SIX13* of chr^RC^ is missing in chr^MLN^. Remarkably, this region of chr^RC^ is flanked with identical sequences (2,763 bp; yellow boxes in Figure 1c) that encode a Tfo1 transposase. The exact same sequence is present as a single copy on chr^MLN^ in Fom001 (the yellow box in Figure 1c). The Tfo1 transposase‐encoding sequences are immediately next to the duplicated regions containing g277 and g14059 in Forc016 and Fom001, respectively (Figure 1c). These observations suggest that loss of the c.300 kb on chr^MLN^ in Fom001 was mediated by recombination between identical Tfo1 transposons. Another transposase‐encoding sequence (2,572 bp) is present at different locations on chr^RC^ and chr^MLN^ (red boxes in Figure 1c).

### 2.4 Functional verification of candidate Forc‐specific virulence genes and the candidate Fom‐specific avirulence gene

To assess whether the candidate virulence genes in Forc016 can turn Fom into a cucumber‐ or watermelon‐infecting strain, one or more virulence genes with their own promoter and terminator were cloned and transformed into Fom005. We managed to clone 9 of the 10 candidate virulence genes. Possibly because multiple homologs of g317 are present in Forc016, PCR amplification of this gene was not successful despite many attempts. Three genes, g287 (homolog of *SIX9*), g288 (unknown protein), and g410 (unknown protein), that are absent in Fom were cloned into one construct; g250 (unknown protein, homolog of g14035) was cloned into one construct; g293 (glucosidase 2 subunit β) and g297 (homolog of *SIX13*) were cloned into the same construct; g310 (unknown protein) and g340 (unknown protein) were cloned into the same construct; and g291 (α‐1,3‐glucosidase) was cloned into a single construct because of its large size. T‐DNA of these five constructs was integrated randomly into the genome of Fom005, and five transformants with correct sequences (confirmed by PCR) from each construct were selected for functional analysis.

To assess whether the unique candidate avirulence gene in Fom, g14035, can turn Forc nonpathogenic to cucumber and/or watermelon, this gene with its native promoter and terminator was cloned and transformed randomly into the genome of Forc016, and four transformants with correct sequences (confirmed by PCR) were selected for further analysis.

**FIGURE 2:**
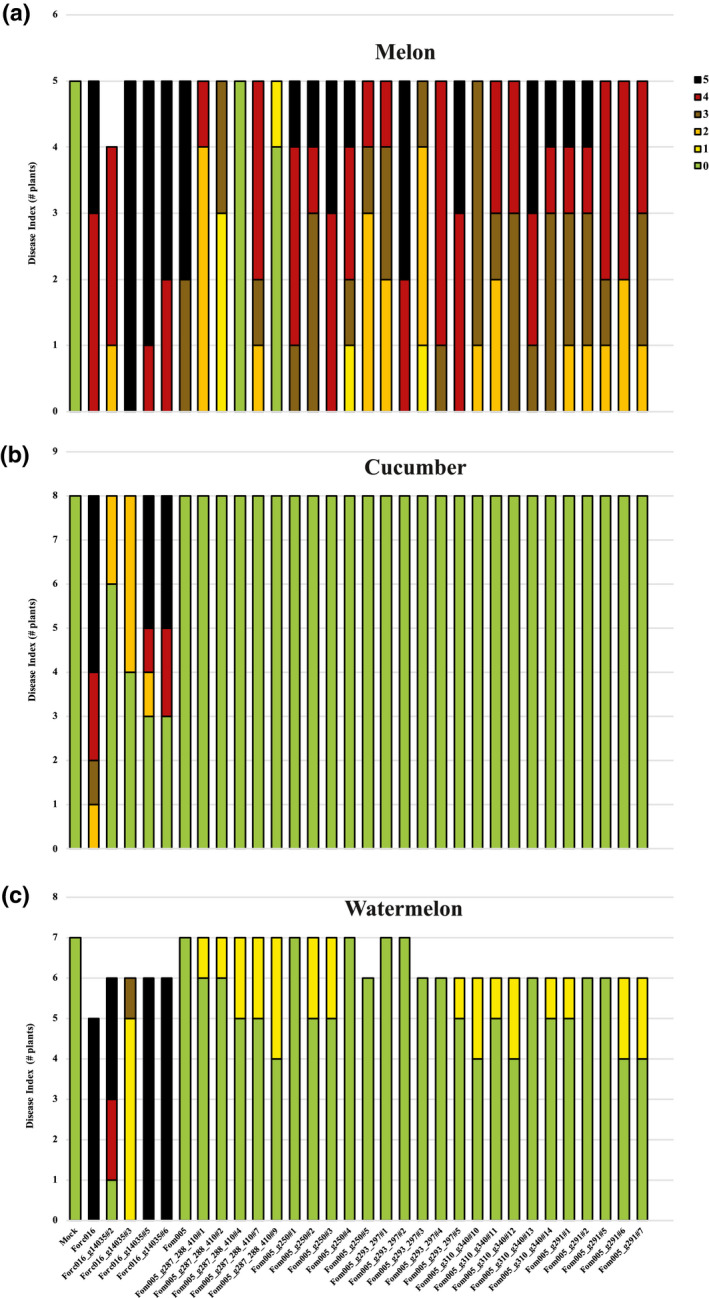
Functional verification of candidate virulence and avirulence genes. Six‐day‐old cucumber and 10‐day‐old melon and watermelon seedlings were inoculated with water (Mock) or indicated strains (10^7^ spores/ml) at 25 °C. Disease index (DI) of infected melon (a), cucumber (b), and watermelon (c) plants were scored 2 weeks after inoculation. Disease symptoms were scored using a disease index ranging from 0 to 5 (0, no symptoms; 1, slight root rot symptoms, only at tip of main root; 2, root rot symptoms and stem lesions visible aboveground; 3, clear root rot symptoms of the entire root system; 4, extensive rot of the entire root system, often with a large lesion extending above the cotyledons, plant very small and wilted; 5, plant completely dead or no green leaves). (a) Except for Fom005_g287_g288_g410#4 and Fom005_g287_g288_g410#9, all Fom005 and Forc016 transformants caused severe disease on melon plants, comparable to the original strains Fom005 and Forc016. (b) No Fom005 transformant was able to cause disease on cucumber plants, while four Forc016 transformants caused less disease on cucumber. (c) No Fom005 transformant could cause disease on watermelon plants, but weak cross‐infection on watermelon plants was observed for some Fom005 transformants. Only slightly increased virulence on watermelon plants was apparent when they were coinoculated with multiple transformants. All Forc016 transformants were still able to cause disease on watermelon plants, albeit reduced virulence was observed for Forc016_g14035#3

All transformants along with the parental strains were tested for virulence in bioassays on cucumber, melon, and watermelon plants. For transformants of Fom005 with putative Forc‐specific virulence genes, except Fom005_g287_g288_g410#4 and Fom005_g287_g288_g410#9, all Fom005 transformants caused severe disease on melon plants, comparable to the parental strain (Figure 2a). Nevertheless, none of the Fom005 transformants was able to cause disease on cucumber or watermelon plants (Figure 2b,c). Consistent with a previous study (van Dam et al., 2016), slight cross‐infection of Fom strains on watermelon plants was observed (Figure 2c).

**FIGURE 3:**
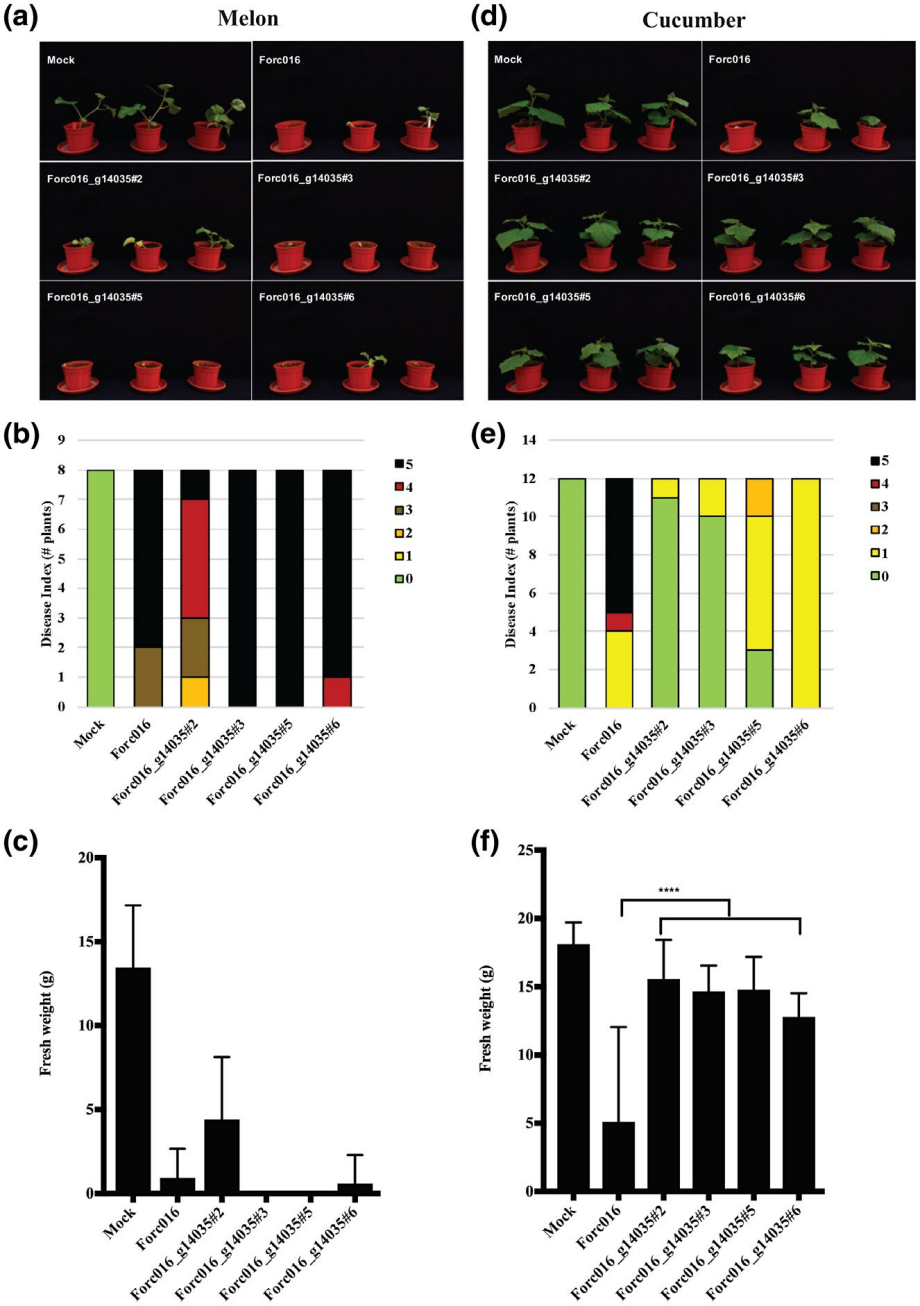
A single gene from *Fusarium oxysporum* f. sp. *melonis* (Fom) turns *F. oxysporum* f. sp. *radicis*‐*cucumerinum* (Forc) nearly nonpathogenic to cucumber plants. Six‐day‐old cucumber and 10‐day‐old melon seedlings were inoculated with water (Mock) or strains (10^7^ spores/ml) at 25 °C. Phenotype (a, d), disease index (b, e), and fresh weight (c, f) of infected melon and cucumber plants, respectively, were scored 2 weeks after inoculation. Disease symptoms were scored using a disease index ranging from 0 to 5 (0, no symptoms; 1, slight root rot symptoms, only at tip of main root; 2, root rot symptoms and stem lesions visible aboveground; 3, clear root rot symptoms of the entire root system; 4, extensive rot of the entire root system, often with a large lesion extending above the cotyledons, plant very small and wilted; 5, plant completely dead or no green leaves). Melon plants infected with any strain were severely diseased (a, b, c), while cucumber plants infected with Forc016 transformants appeared to be healthy. One‐way analysis of variance test (****p < .001) was performed to determine significance of the differences in the fresh weight measurements

Of the Forc016 transformants with the putative Fom‐specific avirulence gene, remarkably two transformants, Forc016_g14035#2 and #3, had almost completely lost their ability to infect cucumber plants, while the other two transformants showed only weak virulence to cucumber plants (Figure 2a). These four transformants were still able to infect melon and watermelon plants, although reduced virulence on watermelon plants was observed for Forc016_g14035#3 (Figure 2b,c). These results were confirmed in a second bioassay performed on cucumber and melon plants for these Forc016 transformants (Figure 3). Cucumber plants infected with these four Forc016 transformants were almost completely healthy, while they were still able to cause disease on melon plants (Figure 3).

In conclusion, candidate Forc‐specific virulence genes were not able to turn Fom pathogenic to cucumber or watermelon plants, while a single gene was able to turn Forc016 nonpathogenic to cucumber plants.

## 3 DISCUSSION

By sequence comparison of predicted genes on pathogenicity chromosomes of Forc and Fom, and bioassays with strains transformed with candidate (a)virulence genes, we showed that a single gene from Fom turns Forc (nearly) nonpathogenic to cucumber plants. This suggests that the encoded protein is recognized by a receptor in cucumber plants and triggers an effective immune response. The mature protein encoded by this gene contains 132 amino acids without any cysteines, and no mimp or other inverted repeats are present upstream of this gene. The finding that the encoded protein, unlike most other avirulence proteins, does not contain cysteine residues should be considered when selecting candidate effector genes. Lack of cysteine residues is not unique to this avirulence protein because avirulence protein AvrM in *Melampsora lini* also lacks cysteine residues (Catanzariti et al., 2006). Gene g250, the homolog of g14035 in Forc at a syntenic position on its pathogenicity chromosome, is expressed during colonization of cucumber, but is remarkably divergent in sequence, suggesting that its product is not recognized by the cucumber immune system. The other homolog of g14035 in Forc, g277, is also expressed during colonization of cucumber. The g277‐ and g14035‐encoded mature proteins differ by two amino acids only. Nonetheless, the protein encoded by g277 is apparently not recognized in cucumber. Apparently, a change in one or two amino acids in the protein can lead to loss of recognition by the cucumber immune system and thus loss of the avirulence function. This is exactly what has been observed before in Fol: a single amino acid change in Avr2 can abolish recognition of Avr2 by I‐2, whereas the virulence function of the protein remains unaffected (Biju et al., 2017; Houterman et al., 2009).

Most plant‐pathogenic fungi have a limited host range (van der Does and Rep, 2007). All plant species outside this host range are nonhosts and are considered to display nonhost resistance. Schulze‐Lefert and Panstruga (2011) suggest that effector‐triggered immunity (ETI) plays a dominant role in nonhost resistance of species closely related to the host, while in distantly related species, pattern‐triggered immunity (PTI) plays a dominant role (Schulze‐Lefert and Panstruga, 2011). Depending on the presence or absence of visual symptoms, nonhost resistance is divided into two types: type I nonhost resistance does not produce any visual symptoms, while type II nonhost resistance is associated with necrosis or cell death. Type I nonhost resistance is similar to PTI, whereas type II nonhost resistance resembles ETI (Mysore and Ryu, 2004).

Previously, we have shown that both Forc and Fom are able to colonize xylem vessels of cucumber, melon, and watermelon roots, indicating that like Forc, Fom is able to overcome constitutive physical and chemical barriers, and PTI at least to some extent (Chisholm et al., 2006; Li et al., 2020). However, further colonization of the upper part of the cucumber and watermelon plants by Fom has not been observed (Li et al., 2020). Because cucumber, melon, and watermelon are closely related plant species and belong to the same family (Cucurbitaceae) and necrosis of infected cucumber and watermelon roots by Fom has been observed (Li et al., 2020), it is most likely that nonhost resistance of cucumber and watermelon to Fom is of type II and ETI plays a dominant role (Mysore and Ryu, 2004; Schulze‐Lefert and Panstruga, 2011).

We initially considered two possibilities for the “late” resistance of cucumber plants against Fom. First, ETI may only be triggered at a late stage, when a secreted effector is recognized by a plant immune receptor. Second, Fom effectors may only partly suppress PTI of cucumber and watermelon plants, and additional effectors or enzymes that are present in Forc are needed. Here, we demonstrate that a single effector gene from Fom, g14035, can turn Forc nonpathogenic to cucumber plants, strongly suggesting that this effector is recognized by an immune receptor in cucumber plants that acts relatively late during infection, conferring nonhost resistance of cucumber to Fom.

How Forc is able to cause disease in watermelon but Fom cannot remains unknown. We consider it most likely that another avirulence gene is present in Fom that can be recognized by watermelon. If this is true, to find this gene an alternative annotation approach of the Fom pathogenicity chromosome will be required, including transcriptome data of Fom‐ and Forc‐infected watermelon.

## 4 EXPERIMENTAL PROCEDURES

### 4.1 Fom001 genome annotation and sequence alignment

We predicted genes in Fom001 (NRRL26406) (Ma et al., 2014) based on RNA‐Seq (Schmidt et al., 2016) and de novo predictions using BRAKER_v2.1.0 (Hoff et al., 2019) with the following flags: ‐‐fungus ‐‐species= “fusarium_oxysporum.” Before gene prediction, repeats and low complexity regions of Fom001 were identified using RepeatMasker v. 4.0.8 (with ‐species “ascomycota”) (A.F.A. Smit, R. Hubley & P. Green RepeatMasker at http://repeatmasker.org).

Chromosome alignments were performed using nucmer (with –maxmatch) from the MUMmer v. 3.23 package (Delcher, 2002).

### 4.2 Forc16‐cucumber RNA‐Seq data reanalysis

To assess quality of sequence reads, FastQC was used (http://www.bioinformatics.babraham.ac.uk/projects/fastqc/). To trim and filter reads, trimmomatic was used. The trimmed reads were aligned to the indexed genome of Forc016 using hisat2 (‐p 2 ‐‐dta ‐x). To remove the nonmapping reads, samtools view (‐Sb ‐F 4 ‐o) was used. Finally, read counts file was generated using featureCounts (‐O ‐t mRNA ‐g ID ‐a). The ideal application with default settings (http://shiny.imbei.uni‐mainz.de:3838/ideal/) was used for differential expression analysis.

### 4.3 Identification of candidate virulence and avirulence genes

To identify candidate virulence and avirulence genes, BLASTN was done against the sequences of chr^MLN^ or chr^RC^ using predicted genes on chr^RC^ or chr^MLN^ as a query fasta. SignalP‐5.0 (http://www.cbs.dtu.dk/services/SignalP/) was used to predict the presence of a signal peptide in the translation products of each of the candidate genes.

### 4.4 Cloning

To express candidate effector genes in Fom005 or Forc016, candidate genes with their original promoter (around 1 kb) and terminator (around 0.5 kb) were amplified from genomic DNA and cloned into pRW1p (Houterman et al., 2008). Candidate genes g287, g288, and g410 were cloned into the same construct; candidate genes g293 and g297 were cloned into the same construct, candidate genes g310 and g340 were cloned into the same construct, and candidate genes g250, g291, and g14035 were cloned into pRW1p separately. Candidate genes were cloned into pRW1p using the HiFi cloning kit (New England Biolabs (UK) Ltd). The primers used to amplify the candidate genes are listed in Table S1.

### 4.5 *Agrobacterium*‐mediated *Fusarium* transformation

To introduce candidate effector genes into Forc016 or Fom005, *Agrobacterium*‐mediated *Fusarium* transformation was performed as previously described (Takken et al., 2004). T‐DNA of each construct was integrated randomly into the genome of Fom005 or Forc016. The presence of the full‐length candidate gene(s) in transformants was confirmed by PCR.

### 4.6 Disease assays

Virulence assays were performed as described previously with some modifications (van Dam et al., 2016). Briefly, spores at 10^7^/ml concentration were used to (co)inoculate seedlings of cucumber (around 6–7 days old), melon (9–10 days old), or watermelon (9–10 days old). For each treatment, six to eight seedlings were inoculated and grown at 25 °C in a greenhouse. The following plant cultivars were used: *Cucumis sativus* ‘Paraiso’, *Cucumis melo* ‘Cha‐T’, and *Citrullus lanatus* ‘Black Diamond’. Two weeks after inoculation, disease symptoms were scored using a disease index ranging from 0 to 5 (0, no symptoms; 1, slight root rot symptoms, only at tip of main root; 2, root rot symptoms and stem lesions visible aboveground; 3, clear root rot symptoms of the entire root system; 4, extensive rot of the entire root system, often with a large lesion extending above the cotyledons, plant very small and wilted; 5, plant completely dead or no green leaves).

## CONFLICT OF INTEREST

The authors declare that they have no competing interest.

## Supporting information


**TABLE S1** Primers used for cloning

**TABLE S2** DNA and protein sequences of putative avirulence and virulence genes

## Data Availability

The data that support the findings of this study are available from the corresponding author upon reasonable request.
