# Supplementary material for: A single gene in Fusarium oxysporum limits host range
Source: Mol Plant Pathol. 2020 Nov 4;22(1):108–16. doi: 10.1111/mpp.13011 (PMC7749751; doi:10.1111/mpp.13011)
Supplement: Supplementary file 1 — TABLE S1 Primers used for cloning [file MPP-22-108-s001.docx]

**Table S1: Primers used for cloning.**

| **Name** | **Sequence** | Ta (℃) |
| --- | --- | --- |
| HiFi_g287_g288_F | acttgtttagaggtaatccttctttCAGGACACTTGGCGTGCTAAG | 69.5 |
| HiFi_g287_g288_R | ggtacgccttggaTTGAAGGCACGCATCGCATTG | 69.5 |
| HiFi_g410_F | gcgtgccttcaaTCCAAGGCGTACCATTACAC | 65.4 |
| HiFi_g410_R | cgttgtaaaacgacggccagtgccaCATCGATCTCGCGTTGCACG | 65.4 |
| HiFi_g250_F | acttgtttagaggtaatccttctttCCATCTGGTGACCCTAGGAATC | 64.5 |
| HiFi_g250_R | tgcagccgtatcgGCATCCCACTGTATTCGTTG | 64.5 |
| HiFi_g317_F | acagtgggatgcCGATACGGCTGCAAAGTGTG | 65.6 |
| HiFi_g317_R | cgttgtaaaacgacggccagtgccaACTATGCTCCGAATCTTGCC | 65.6 |
| HiFi_g293_F | acttgtttagaggtaatccttctttAGGAGTTGGAGAGCCAGAAC | 67.6 |
| HiFi_g293_R | atcaaatcccacgTAGAGGGTGGAGCAGAGAGC | 67.6 |
| HiFi_g297_F | ctccaccctctaCGTGGGATTTGATCGAGACTAG | 63.1 |
| HiFi_g297_R | cgttgtaaaacgacggccagtgccaGAGTATCAGTAGTAACATCGTGAATG | 63.1 |
| HiFi_g310_F | acttgtttagaggtaatccttctttCTTCTATCTAGTTCTGGCCTAC | 62 |
| HiFi_g310_R | gctcaccagctgCTGATACTCTGCTCTATGCG | 62 |
| HiFi_g340_F | agcagagtatcagCAGCTGGTGAGCTATCATAC | 61.1 |
| HiFi_g340_R | cgttgtaaaacgacggccagtgccaCACCAAGAAGAATATCCACG | 61.1 |
| HiFi_g291_F | acttgtttagaggtaatccttctttCGAATTGCCACACCAAAGGAG | 67 |
| HiFi_g291_R | cgttgtaaaacgacggccagtgccaTCGAGAGGTGGTTACGCACAAAG | 67 |
| HiFi_g14035_F | acttgtttagaggtaatccttctttCGACTTACACACCTGCGAATAC | 64 |
| HiFi_g14035_R | cgttgtaaaacgacggccagtgccaATCTGGTGACCCTAGGAATC | 64 |

Ta: annealing temperature.
